# Organic ultraviolet filters in nearshore waters and in the invasive lionfish (*Pterois volitans*) in Grenada, West Indies

**DOI:** 10.1371/journal.pone.0220280

**Published:** 2019-07-24

**Authors:** Ryan A. Horricks, Sarah K. Tabin, Jonnel J. Edwards, John S. Lumsden, David P. Marancik

**Affiliations:** 1 Department of Pathobiology, Ontario Veterinary College, University of Guelph, Guelph, ON, Canada; 2 Department of Pathobiology, School of Veterinary Medicine, St. George’s University, True Blue, Grenada, West Indies; Trent University, CANADA

## Abstract

Sunscreens and other personal care products use organic ultraviolet (UV) filters such as oxybenzone, 4-methylbenzylidene camphor, Padimate-O, and octyl methoxycinnamate to prevent damage to human skin. While these compounds are effective at preventing sunburn, they have a demonstrated negative effect on cells and tissues across taxonomic levels. These compounds have a relatively short half-life in seawater but are continuously re-introduced via recreational activities and wastewater discharge, making them environmentally persistent. Because of this, testing seawater samples for the presence of these compounds may not be reflective of their abundance in the environment. Bioaccumulation of organic ultraviolet filters in a high-trophic level predator may provide greater insight to the presence and persistence of these compounds. To address this, the present study collected seawater samples as well as muscle and stomach content samples from the invasive Pacific lionfish (*Pterois volitans*) in the nearshore waters of Grenada, West Indies to examine the use of lionfish as potential bioindicator species. Seawater and lionfish samples were collected at four sites that are near point sources of wastewater discharge and that receive a high number of visitors each year. Samples were tested for the presence and concentrations of oxybenzone, 4-methylbenzylidene camphor (4-MBC), Padimate-O, and octyl methoxycinnamate (OMC) using liquid chromatography-mass spectrometry. Oxybenzone residues were detected in 60% of seawater samples and OMC residues were detected in 20% of seawater samples. Seawater samples collected in the surface waters near Grenada’s main beach had oxybenzone concentrations more than ten times higher than seawater samples collected in less frequently visited areas and the highest prevalence of UV filters in lionfish. Residues of oxybenzone were detected in 35% of lionfish muscle and 4-MBC residues were detected in 12% of lionfish muscle. Padimate-O was not detected in either seawater or lionfish samples. No organic UV filters were detected in lionfish stomach contents. Histopathologic examination of lionfish demonstrated no significant findings attributed to UV filter toxicity. These findings report UV filter residue levels for the first time in inshore waters in Grenada. Results indicate that lionfish may be bioaccumulating residues and may be a useful sentinel model for monitoring organic ultraviolet filters in the Caribbean Sea.

## Introduction

Personal care products (PCPs) are of increasing concern as environmental contaminants due to their widespread use and potential toxicity. Many of the compounds used in PCPs are persistent, bioactive, bioaccumulative, and endocrine-disrupting [1]. Organic ultraviolet (UV) filters are used in PCPs, including sunscreens, to prevent skin damage and have emerged as important contaminants of aquatic ecosystems. Most notably, oxybenzone (BP-3), octyl methoxycinnamate (OMC), Padimate-O, and 4-methylbenzylidene camphor (4-MBC) are organic UV filters typically found in sunscreens that are released incidentally to coral reef areas [2]. These, and other UV filters can enter the water directly from recreational activities or indirectly from waste water and sewage [3–7].

All four of the compounds; oxybenzone, 4-MBC, OMC, and Padimate-O, have demonstrated negative effects on cells and tissues in both vertebrate and invertebrate species. The contaminants disrupt estrogen signaling pathways, induce reproductive pathologies, and reduce reproductive fitness in fish [8,9] and are directly toxic to invertebrates across trophic levels including the Mediterranean mussel (*Mytilus galloprovincialis*), the purple sea urchin (*Paracentrotus lividus*), mysid shrimp (*Siriella armata*) [10], *Stylophora pistillata* coral larvae [11]), and the protozoan *Tetrahymena thermophila* [12].

The quantity of organic UV filter residues that enter the world’s oceans are currently unknown. Using an average dose application of 2mg cm^-2^ one group estimated that 4000–6000 tons of sunscreen wash off people in coral reef areas annually [13], while another provided an estimate of 6000–14000 tons [11]. Given the potential for UV filter residues to negatively affect the organisms living in and around coral reefs, there is a distinct benefit in identifying risks to local ecosystems through the monitoring of contaminant levels. This is especially true in areas of already stressed or declining reef conditions, around areas of potential wastewater discharge, and near relatively high-density beaches [14] and tourist areas that receive a substantial number of visitors on a regular basis. Previous studies that have quantified UV filters in aquatic environments have detected variable concentrations ranging from none up to 1.4 parts per million depending on the region and sampling conditions [11,15]. The relatively short half-life of these contaminants in water and the possible vulnerability of testing results to environmental conditions suggests that testing seawater for the presence of the compounds may not be reflective of their levels in marine life. While these contaminants typically have short half-lives, they can be considered environmentally persistent due to their frequent re-introduction to the aquatic environment [1,16].

Previous studies have found detectable ranges of UV filters in invertebrates [17], mammals [18], and fish [19]. Many of the organic UV filters found in PCPs are bioaccumulative [2] and will biomagnify as they ascend trophic levels [20]. This suggests that predatory species may be useful for characterizing the magnification potential of UV filters and act as bioindicators for monitoring trends in environmental levels.

Lionfish (*Pterois volitans*) may represent a promising sentinel model to monitor environmental UV filters within the Caribbean Sea. Since their introduction to the western Atlantic in 1985, populations of the invasive lionfish have greatly increased in abundance and distribution in the Caribbean despite substantial containment efforts [21]. Lionfish are voracious predators whose diet primarily consists of teleost species and crustaceans [22] and as a high trophic level predator, lionfish have the potential to bioaccumulate organic UV filters in their tissues. Lionfish are routinely culled by local dive shops and fishermen and represent an emerging food source in the Caribbean, which provides a consistent supply of samples while preserving endemic fish species. Given their continued presence and sedentary nature on Caribbean coral reefs, voracious appetites, relatively high trophic level, and availability, the present study explored the feasibility of using lionfish as sentinel model for the detection of the organic UV filters oxybenzone, OMC, Padimate-O, and 4-MBC. Additionally, examination of muscle from lionfish will examine potential human exposure to contaminants through the food chain.

To date, the presence of UV filters within the inshore waters of Grenada have not been investigated. The present study collected wild lionfish adjacent to and away from relatively high-density tourist beaches in Grenada and tested these samples for the presence of four commonly occurring organic UV filters. The high-density tourist area of the Grand Anse region of Grenada used in the present study represents an increasingly popular recreational and cruise ship destination for beach-goers, swimmers, and divers. It also is a recently designated marine protected area with important coral and fish habitat in addition to ongoing coral restoration projects. Comparison of UV filter concentrations in lionfish and water samples in the present study provides baseline data for ongoing monitoring efforts in Grenada and explores the utility of lionfish as sentinel models of environmental contamination.

## Materials and methods

### Sample collection

Sample collection was performed under permit (001) from the Grenada Ministry of Agriculture, Lands, Forestry, and the Environment and with approval by St. George’s University Institutional Animal Use and Care (IACUC) Committee (18011-R).

**Fig 1:**
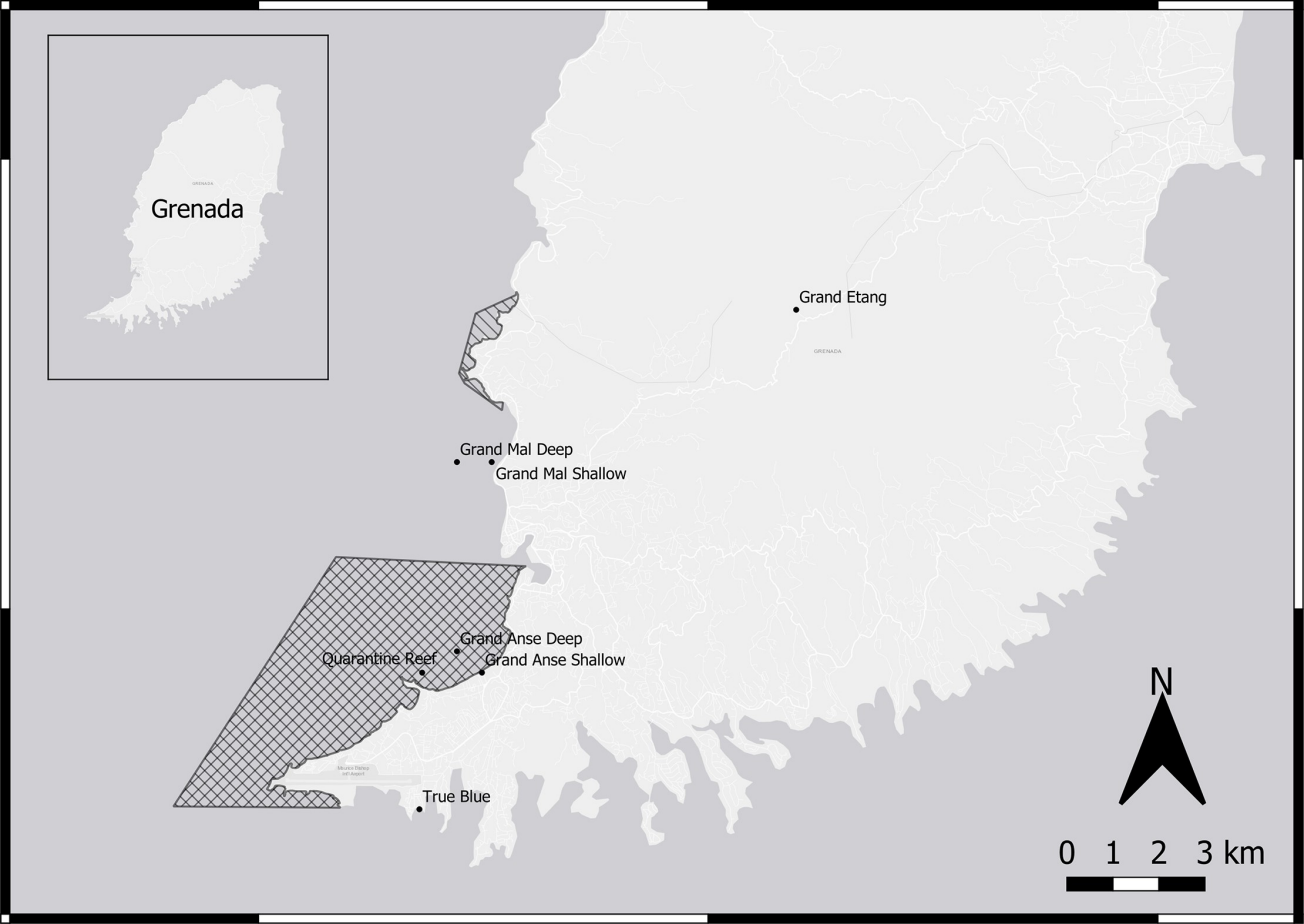
Locations for sampling sites in Grenada, West Indies. Boundaries of the Molinere-Beasejour marine protected area (hashed) and proposed Grand Anse protected area (crossed) are indicated.

Seventeen lionfish (ten females, seven males) and five 200mL seawater samples were collected from the nearshore waters of Grenada from September 2017 to March 2018. Fish were collected by SCUBA divers at three different regions on the West and Southwest coasts of Grenada using spear poles at depths of 3-30m (Table 1, Fig 1) as part of the regional lionfish culling program. Two seawater samples and five lionfish were collected on September 5^th^, 2017 in Grand Anse Bay and six lionfish were collected on February 13^th^, 2018 at Quarantine Reef. These sites are adjacent to the main recreational swimming areas in Grenada (Grand Anse Beach) that experience a consistent number of beach-goers and are down current from the relatively densely populated town of St. George’s (Fig 1). An additional surface water sample was collected off Grand Anse Beach on February 13^th^. On March 1^st^, 2018 six lionfish and two seawater samples were collected at Grand Mal, located North and up-current of Grand Anse Beach and the town of St. George’s (Fig 1). Two negative controls were utilized to ensure quality assurance for collection, transportation, and assay techniques. One tilapia (*Oreochromis* sp.) was collected by hand net from the Grand Etang Lake, located within the National Park. Swimming is not permitted at Grand Etang Lake and there is no overt industrial or residential activity in the surrounding area. Euthanasia was performed via immersion in 200mg mL^-1^ tricaine methanesulfonate (MS-222, Sigma-Aldrich, St. Louis, MO, USA). One seawater sample was collected at True Blue Bay on March 1^st^, 2018, which has relatively low, and most often no recreational activity.

**Table 1:** Site names, GPS coordinates, and sample type collected in the nearshore waters of Grenada, West Indies.

Site Name	Sample(s) Collected	GPS Coordinates
Grand Anse Shallow	Seawater	12° 1ʹ 31.00″ N 61° 45ʹ 45.00″ W
Grand Anse Deep	Seawater, *P*. *volitans*	12° 2ʹ 01.00″ N 61° 45ʹ 32.00″ W
Quarantine Reef	*P*. *volitans*	12° 1ʹ 28.00″ N 61° 46ʹ 17.00″ W
Grand Mal Shallow	Seawater, *P*. *volitans*	12° 3ʹ 59.00″ N 61° 45ʹ 27.00″ W
Grand Mal Deep	Seawater, *P*. *volitans*	12° 3ʹ 46.00″ N 61° 45ʹ 34.00″ W
True Blue Shallow	Seawater	11° 59ʹ 55.00″ N 61° 46ʹ 19.00″ W
Grand Etang	*Oreochromis* sp.	12° 05ʹ 46.00″ N 61° 41ʹ 48.00″ W

Seawater was collected at the surface and at depth by SCUBA divers using sterile 200mL Nalgene Laboratory containers (Thermo Fisher, Rochester, NY, USA) and transported on ice for ≤ 4 hours until storage at -80°C. Seawater samples were collected in plastic bottles for safety and ease of transport to the laboratory. Surface seawater samples were collected approximately 20cm below the surface of the water and deep seawater samples were collected at the maximum depth of the dive (25m at Grand Mal and 12m at Grand Anse).

Lionfish were placed on ice after collection and brought to the Aquatic Animal Medicine Research Laboratory at St. George’s University, School of Veterinary Medicine (True Blue, Grenada) for tissue collection within 3 hours. One muscle sample (5.09g ± 0.62) was removed from the lateral flank of each fish and frozen at -80°C. Stomachs were removed and the stomach contents (4.33g ± 1.55) from fish with digesta present were pooled by site. Extracted muscle and stomach contents were sent to Jupiter Environmental Laboratories (Jupiter, FL, USA) for liquid chromatography-mass spectrometry (LC-MS) analysis. Gill, heart, liver, spleen, kidney, and gonad tissues were collected for histological examination. Histological samples were fixed in 10% buffered formalin for ≥ 48 hours before being routinely processed, sectioned at 5μm, and stained with hematoxylin and eosin. Tissues were examined for signs of cellular changes including but not limited to necrosis, nuclear pleomorphism, megalocytosis, neoplasia, gonadal atresia, and signs of tissue regeneration, fibrosis, or replacement by other cell types.

### Sample extraction for LC-MS

Lionfish muscle and stomach samples were pulped and 20μg kg^-1^ D5-oxybenzone (Sigma Aldrich) was added as a surrogate for calibration, standardization, and quality control. Following surrogate addition, methanol (Fisher Scientific, Tampa, FL, USA) was added to the sample. The extracts were sonicated for one hour and subsequently spun down and injected for LC-MS analysis. Surrogate was added to seawater samples and the analytes were extracted with methylene chloride (Fisher Scientific). Seawater extracts were evaporated until dry and re-suspended in methanol for LC-MS analysis.

Samples were analyzed in triplicate on an AB Sciex Qtrap 5500 coupled to a Symbiosis Pico (Spark Holland B.V., Emmen, NL). The LC utilized the conditions in Table 2 using HPLC water with 0.15% formic acid (TCI, Tokyo, Japan) as mobile phase A and methanol with 0.1% formic acid as mobile phase B. The LC columns used were a Luna 5μ C18 30 x 2mm (Phenomenex, Torrence, CA, USA) as a pre-column and a Kinetex 1.7μm EVO C-18 100Å 50 x 2.1mm at 40°C. Analysis of standards was performed using 11.5μg kg^-1^ (tissue) or 0.04μg L^-1^ (seawater) oxybenzone (Sigma Aldrich), Padimate-O (Accustandard, New Haven, CT, USA), 4-Methyl-Benzylidene Camphor (Accustandard), and Octyl-methoxycinnamate (Accustandard). Standards were fit with a linear function with R^2^ ≥ 0.99. Percent recovery was reported between 107–121%. The ion transitions and energies are shown in Table 3. The curtain gas was set to 20 PSI with medium collision gas, ion spray voltage of 500, temperature of 400°C, ion source gasses one and two set to 50 PSI.

**Table 2:** Liquid chromatography conditions.

Time	% A	% B
0:01	95	5
0:50	30	70
10:00	2	98
14:00	2	98
14:30	95	5
16:50	95	5

**Table 3:** Ion transitions and energies used for liquid chromatography.

Q1 (Da)	Q3 (Da)	Compound	Declustering Potential (V)	Collison Energy (V)	Collision Cell Exit Potential (V)
256	76.9	4-Methyl-Benzylidene Camphor 1	96	83	12
256	105	4-Methyl-Benzylidene Camphor 2	96	41	6
229.1	151	Oxybenzone 1	130	30	10
229.1	104.9	Oxybenzone 2	130	35	12
292	162	Octyl-methoxycinnamate 1	55	47	20
292	134	Octyl-methoxycinnamate 2	55	47	22
279	152	Padimate O 1	55	47	12
279	135	Padimate O 2	55	69	9
279	167	Padimate O 3	55	25	12
235.2	152.1	Oxybenzone D5 1	123	47	12
235.2	110	Oxybenzone D5 2	123	47	12

### Statistical analysis

Logistic regression was used to test for the effect of site on the detection of environmental contaminants in lionfish muscle and seawater samples. A Kruskal-Wallis test was used to test for the effect of site on contaminant concentration in both lionfish muscle and seawater samples. A Spearman’s correlation was used to compare contaminant concentrations with lionfish length and weight. All statistical analyses were conducted using R Version 3.4 [23] with a significance level of α = 0.05.

## Results

UV filter residues were detected in 3/5 (60%) water samples. Oxybenzone was detected in water samples at a mean concentration of 0.046μg L^-1^ ± 0.067 (mean ± SD). This included detection of the highest concentration in the shallow seawater sample from Grand Anse Beach (0.123μg L^-1^) which was over seventeen times higher than those found in the deep seawater sample from Grand Anse Bay (0.00659μg L^-1^) and the shallow seawater sample from Grand Mal (0.00708μg L^-1^) (Table 4). OMC was detected in the deep seawater sample from Grand Anse Bay at a concentration of 0.00834μg L^-1^ (Table 4). Padimate and 4-MBC were not detected in any of the seawater samples (Table 4). The water sample collected at True Blue Bay tested negative for all four contaminants.

**Table 4:** Average concentrations (μg kg^-1^ or μg L^-1^) of organic ultraviolet filters in lionfish muscle and seawater samples collected in Grenada, West Indies.

Sample	Oxybenzone	4-MBC	OMC	Padimate
*Grand Anse Bay*				
Surface (beach) water	0.123μg L^-1^	X	X	X
Deep water	0.007μg L^-1^	X	0.008μg L^-1^	X
Lionfish muscle	2.90μg kg^-1^ ± 3.62	2.11μg kg^-1^ ± 0.71	X	X
*Quarantine Reef*				
Surface water	X	X	X	X
Deep water	X	X	X	X
Lionfish muscle	0.12μg kg^-1^ ± 0.01	X	X	X
*Grand Mal*				
Surface water	0.007μg L^-1^	X	X	X
Deep water	X	X	X	X
Lionfish muscle	0.17μg kg^-1^	X	X	X
*True Blue*				
Surface water	X	X	X	X

X–Indicates that contaminants were not detected or detected below laboratory limits.

On average, lionfish weighed 290.64g ± 228.98 (mean ± SD) and were 26.21cm ± 6.31 (mean ± SD) long. Histopathologic examination revealed no significant microscopic changes in gill, heart, liver, spleen, kidney, or gonad that would be attributed to environmental toxicity. The tilapia used as a negative control weighed 45.0g and was 17.0cm long and was negative for all four contaminants (Table 4).

UV filter residues were detected in muscle from 6/17 (35%) lionfish (Table 4). Oxybenzone was detected in four (24%) lionfish (two females and one male) at concentrations of 1.46μg kg^-1^ ± 2.67 (mean ± SD). This included one fish collected at Grand Anse, two fish at Quarantine Reef, and one fish at Grand Mal. 4-MBC was detected in two (12%) lionfish (one female and one male) collected at Grand Anse at concentrations of 2.11μg kg^-1^ ± 1.00 (mean ± SD). One fish (male) collected at Grand Anse tested positive for both oxybenzone and 4-MBC. This fish had the highest concentrations of both environmental contaminants detected in its tissues (Table 4). No OMC or Padimate were detected in any of the lionfish muscle samples.

Pooled stomach contents present in 3/6 (50%) lionfish from Grand Anse Bay, 4/6 (67%) lionfish from Quarantine Reef, and 2/6 (33%) lionfish from Grand Mal were grossly identified as a partially digested teleost fish, shrimp, and crab. No sunscreen residues were detected in any pooled lionfish stomach contents.

There were no significant effects of site on the detection (p = 0.165) or the concentration (p = 0.573) of oxybenzone in muscle tissues. There were no significant correlations found between oxybenzone levels and fish weight (p = 0.356) or length (p = 0.283). The relatively low sample size used in the present study does not provide high statistical confidence.

## Discussion

The goal of the present study was to examine the presence of four common UV filter residues in the inshore waters of Grenada and to explore the use of lionfish as sentinel models to monitor trends in levels of fauna. Results indicate, for the first time, that oxybenzone, 4-MBC, and OMC are present within coral reef habitats in Grenada with relatively higher levels of oxybenzone associated with the shallow water near Grand Anse Beach. Detection of residues in lionfish indicate their utility as a bioindicator species to monitor persistent contaminant levels within reef systems. To date, there is no evidence to suggest that UV filter residue levels in lionfish muscle pose a human health concern as lionfish represent an emerging food source in the Caribbean. There were no observed trends associating UV filter residue levels with the sex or morphometrics of fish, however, the sample size was relatively small and there was low statistical confidence.

Absolute coral cover in the Caribbean has decreased dramatically over the past four decades with some temporal variation in both the rate and amount of coral decline [24,25]. The variability between areas is strongly influenced by local factors including the impacts of high-density tourism [24,25]. This demonstrates that while tourism comprises a growing portion of Grenada and many other Caribbean nations’ gross domestic product, it also has the potential to negatively impact natural resources [24,26]. Contamination of reef systems with UV filter residues from recreational use and effluent water represents a growing threat to ecosystems that may rise as tourism continues to increase in the region [11]. Development of baseline UV filter residue levels in local reef systems provides a means to monitor toxicant levels over time and assess thresholds for sustainable tourism and use of inshore reef environments.

The four organic UV filters tested for in the present study were chosen because they have been historically used in PCPs and have a wide range of demonstrated negative effects in fish and mammals. This includes action as reproductive toxicants [11] with negative effects on reproductive fitness [27–29] and direct injury to cells and tissues [30–33]. No overt changes were observed on a histopathologic level in tissues of necropsied lionfish. This may be a reflection of the relatively low residue concentrations detected, but histology may also not be a sensitive biomarker of toxicologic effects of UV filter residues. A previous study in zebrafish showed changes in hormonal gene transcripts associated with oxybenzone doses of 84μg L^-1^ but gonadal histology was not affected, even when challenge doses reached > 400 μg L^-1^ [34]; however, little is known about the response of lionfish to toxicants. Validation of gene expression profiles and further biochemical and subcellular studies are needed to interpret the effect these residues may have on lionfish as well as endemic marine fish.

In the present study, oxybenzone was the most prevalent organic UV filter. This is not surprising given that it is the most common organic UV filter used in commercially available sunscreens and other PCPs [35,36]. The use of oxybenzone in PCPs is so prevalent that it was detected in 98% of human urine samples in a survey of the US population [37]. Although the low sample size in this study limited statistical comparisons between sites, there was a trend towards higher prevalence and concentration of oxybenzone in seawater and lionfish associated with the more populated and visited areas of Grenada. Oxybenzone was present in surface and deep seawater samples and four lionfish collected from Grand Anse Bay and Quarantine Reef, which are close to Grand Anse Beach and the relatively densely populated area of St. George’s. The highest concentrations of oxybenzone were detected in the surface water samples and lionfish collected closest to Grand Anse Beach. One fish from this area also had 4-MBC present in its musculature. Oxybenzone was also detected in the surface seawater sample and one lionfish from Grand Mal. Although this area is up-current of Grand Anse and St. George’s, it is down current of several small villages along the coastline and near popular snorkeling and diving areas. These findings indicate that coral reef habitat along the Western coast of Grenada may be at risk of contamination by UV filters, most notably oxybenzone, but potentially 4-MBC and OMC as well. This is especially noteworthy as these areas represent important habitat within the National Marine Protected Parks. Further monitoring over time and with a larger sample size may provide more perspective as to the relative trends and associated risks these compounds pose to fauna in Grenada. Padimate was not present in any muscle or seawater samples. Due to its carcinogenic and mutagenic effects Padimate-O has been eliminated in most commercially available sunscreens [33,38] which may explain its lack of detection.

The degree at which residues are accumulating in lionfish muscle through ingestion of contaminated prey species or through direct exposure from the water is unknown. Both routes have been shown to be important for bioaccumulation and biomagnification of sunscreen residues in fish [34,39]. No UV filter residues were detected in stomach contents to suggest ingestion of contaminated prey in this study. Bioaccumulation of substances in aquatic species can be affected by sex, reproductive status, size, body lipid content, excretion, and geographical location where the sample was collected [7,19,40,41]. The octanol-water partition coefficient (*K*_*OW*_) is typically used to indicate the hydrophobicity of a substance [42,43] and the log *K*_*OW*_ provides an estimate of the likelihood that it will disassociate from water and bioaccumulate in tissue [42]. Highly lipophilic and poorly biodegradable UV filters have a log *K*_*OW*_ of 4–8 [7] and a log *K*_*OW*_ value of 5 or greater is typically used to assess the potential for bioaccumulation [42]. Of the organic UV filters examined in the present study, oxybenzone, 4-MBC, and Padimate have log *K*_*OW*_ values of 3.79, 4.95 and 5.77, respectively. OMC has a relatively high log *K*_*OW*_ value of 6.1 and would be expected to be present in muscle samples as it was detected in seawater samples. Whether a lack of OMC detection in lionfish is a reflection of the low sample size, tissues collected for testing, or other factors requires further investigation. Sampling lionfish tissues with higher lipid content (i.e. the liver) may allow for the detection of UV filters or their metabolites which could be used to assess their potential to bioaccumulate in lionfish.

The relative ease and frequency that lionfish are culled in the Caribbean provides ample opportunity to test fish for UV filter contamination while preserving endemic species; however, the higher percentage of water samples positive for UV filters compared to lionfish indicates that water remains a valuable testing substrate to confirm the presence of UV filters in aquatic environments. The cost of LC-MS analysis remains prohibitive for screening populations using large sample sizes. Examining the effects of pooled tissue samples and collection of tissue with higher lipid content [41] may allow for easier detection of organic UV filters in future work. Future field studies will examine potential seasonality and environmental effects on UV filter residue concentrations in water and lionfish tissue. Lionfish may also serve as a valuable laboratory model to elucidate the dynamics of bioaccumulation and biomagnification within a higher trophic fish species. This may help explain the overall lower prevalence of 4-MBC and OMC in lionfish tissue and whether this is associated with differences in exposure or bioaccumulation of each specific compound.
